# Using State Space Grids for Modeling Temporal Team Dynamics

**DOI:** 10.3389/fpsyg.2019.00863

**Published:** 2019-04-24

**Authors:** Annika L. Meinecke, Clara S. Hemshorn de Sanchez, Nale Lehmann-Willenbrock, Claudia Buengeler

**Affiliations:** ^1^Department of Industrial/Organizational Psychology, Institute of Psychology, University of Hamburg, Hamburg, Germany; ^2^Department of Human Resource Management and Organization, Institute of Business, University of Kiel, Kiel, Germany

**Keywords:** team science, dynamic systems theory, state space grids, team process dynamics, interaction analysis

## Abstract

We outline the potential of dynamics systems theory for researching team processes and highlight how state space grids, as a methodological application rooted in the dynamic systems perspective, can help build new knowledge about temporal team dynamics. Specifically, state space grids visualize the relationship between two categorical variables that are synchronized in time, allowing the (team) researcher to track and capture the emerging structure of social processes. In addition to being a visualization tool, state space grids offer various quantifications of the dynamic properties of the team system. These measures tap into both the content and the structure of the dynamic team system. We highlight the implications of the state space grid technique for team science and discuss research areas that could benefit most from the method. To illustrate the various opportunities of state space grids, we provide an application example based on coded team interaction data. Moreover, we provide a step-by-step tutorial for researchers interested in using the state space grid technique and provide an overview of current software options. We close with a discussion of how researchers and practitioners can use state space grids for team training and team development.

## Introduction

Team researchers agree that teams are inherently dynamic in nature (e.g., Cronin et al., 2011; Herndon and Lewis, 2015; Waller et al., 2016). Teams are often referred to as complex dynamic systems that evolve and change over time as they adapt to new and changing task demands, or as members leave or join the team (Arrow et al., 2000; McGrath et al., 2000; Kozlowski and Ilgen, 2006). Because teams are comprised of independent actors that interact over time, the evolution of teams is non-linear and highly dynamic (e.g., Guastello and Liebovitch, 2009). A recent review of the literature on teams as complex and dynamic systems emphasizes the need for team research to embrace methods that can account for this complexity and dynamism at the core of team processes (Ramos-Villagrasa et al., 2018).

Yet, existing research is often based on simplified theoretical models that do not appropriately account for dynamic team processes. For example, McGrath (1964) seminal work emphasized the central role of team processes as the underlying mechanism by which team members combine their individual resources to resolve team task demands. Yet, team processes are often treated as if they were “frozen” in a mediation box (Kozlowski, 2015), rather than accounting for the complex temporal interaction dynamics at the core of most team processes (e.g., Lehmann-Willenbrock and Allen, 2018).

In this paper, we draw from dynamic systems theory (e.g., Thelen and Smith, 1998) to address the challenge of adequately conceptualizing and operationalizing temporally embedded team processes. Specifically, we propose to study how teams evolve and mature in organizations by showcasing how state space grids (SSGs, Lewis et al., 1999; Hollenstein, 2013) as a methodological application rooted in dynamics system theory can capture and advance our understanding of complex team temporal dynamics. SSGs were originally used by developmental psychologists to study how developmental states occur in real time and how, over time, interpersonal patterns form and stabilize (Hollenstein, 2007, 2013). We argue that team science can greatly benefit from this approach. We discuss the benefits of the dynamic systems perspective for team science and illustrate how SSGs can trigger novel insights into team evolution and maturation, address previous methodological shortcomings, and pave the way for innovative team feedback and intervention practices.

In sum, the aim of our paper is to (a) provide a discussion of how dynamic systems theory can advance our understanding of non-linear processes unfolding in groups and teams^1^, (b) give an in- depth, step-by-step tutorial of how to use the SSG technique to empirically test ideas derived from dynamic systems theory, and (c) outline the benefits of SSGs for both team research and team development. To illustrate the approach, we present sample SSGs generated from coded team interactions.

## Dynamic Systems Theory

A dynamic system is defined as a collection of elements that change over time (Alligood et al., 1996; Thelen and Smith, 1998). As group and team researchers, we are interested in the human domain and therefore focus on groups of individuals in terms of such dynamic systems (see also McGrath et al., 2000). In doing so, we regard groups as *open* rather than closed systems because they are embedded in and interact with their surrounding environment, rather than being isolated from it (Arrow et al., 2000; Marrone, 2010). Of note, dynamic systems theory is not limited to the study of humans. It originated from the fields of physics and mathematics and was later transferred to biological and psychological research (for a more detailed discussion of the foundations and history of dynamics systems theory, see Guastello and Liebovitch, 2009 as well as Thelen and Smith, 1998). In the following, we will outline the basic assumptions underlying dynamic systems approaches and illustrate them with examples from both developmental psychology—the field of psychology in which the dynamic systems perspective is most strongly represented —and team research. We acknowledge here that our outline of dynamics systems theory comprises only its basic structure but that there is much more to explore about the dynamics systems perspective and how it can help to shed new light on how teams evolve and mature over time. We encourage interested readers to follow up with the seminal work of Arrow et al. (2000) who have described teams as complex, adaptive systems in more detail.

The central tenet of dynamic systems theory is that a system (e.g., an individual, a dyad, or a team) can only be in one state at any given moment in time, although several states are available (Thelen and Smith, 1998). For a team researcher, such states may be specific behaviors but they could also represent emotional, affective, or cognitive elements. A system is usually characterized by a certain degree of variability, meaning that it moves from state to state. The change from one state to another describes the *dynamics* of a system. These dynamics are typically messy, difficult to predict, and non-linear in nature. Despite this inherently dynamic perspective, systems do not operate randomly but tend to stabilize in certain states. Thus, over time stable and recurrent patterns emerge. This idea of self-organization or emergence (a term more familiar to team science; Kozlowski, 2015; Waller et al., 2016) “is at the heart of any dynamic systems approach” (Hollenstein, 2013, p. 3; see also Lewis, 2000).

Self-organization in dynamic systems theory is largely seen as a bottom-up process. Higher-order patterns that are characteristic for a system emerge from interactions among lower-order elements represented by individual transitions between states. This process of emergence is often spontaneous and thus challenges traditional ideas of determinism (Lewis, 2000). It is important in this context that dynamic systems theory rather functions as a meta-theoretical framework (Hollenstein, 2013). It is not bound to a specific time frame, but provides a flexible account for understanding the changes of dynamic systems. Dynamic systems can change and stabilize over the course of minutes, weeks, months, or years. Depending on the specific research question at hand and the phenomenon to be examined, a suitable time scale must be selected to observe the dynamics of the particular system. Further adding to its complexity, the dynamics systems perspective assumes that change is hierarchically nested in time (Granic, 2005). This means that patterned structures at a higher level also have a top-down effect in that they shape and constrain interactions among lower-order elements.

To make these assumptions more tangible, we can extrapolate from examples from developmental psychology (e.g., Lewis, 2000; Hollenstein, 2011). In this line of research, lower-order dynamics are often studied in real time at the moment-to-moment (micro) level. For instance, the dynamic systems perspective can help to understand how emotional development unfolds over time (for an edited volume see Lewis and Granic, 2000). At the micro level, emotional states are fast and fleeting and can change within seconds. Over the course of minutes or hours, however, they can persist and transform into more stable moods. These moods, in turn, impact real-time emotional states. It is less likely that we experience instantaneous joy and happiness when we are currently in a bad mood. Through a developmental lens, such recursive patterns can be traced even further. A multiplicity of factors, such as the environment in which we grow up or our temperament, influence which emotional experiences repeatedly solidify and expand into moods. In the long run, often over the course of years, these experiences shape our personality. Personality then has further top-down effects and influences how we behave in and evaluate certain (emotional) situations (see also Hollenstein, 2013).

Transferred to team research, dynamic systems theory can help us understand how moment-to-moment interactions among team members may result in repeating and stable patterns of behavior, such as those that lead to the development of group norms (e.g., norms for turn taking during an organizational meeting). These group norms may well restrict the team members’ behavior during subsequent team meetings. Thus, dynamic systems theory postulates causal processes both *within* and *between* time scales (Hollenstein, 2013). Next, we briefly outline the key terminology associated with dynamic systems theory before introducing SSGs as a method for applying dynamic systems theory to the study of team evolution and maturation.

### State Space, Attractors, Repellors, and Phase Transitions

As a system transitions from one state to another it moves within a specific space. This space is defined by the range of all possible states and is referred to as the *state space* (Hollenstein, 2007). As outlined above, dynamic systems tend to stabilize such that they rarely explore or “visit” the full range of possible states in the state space. In other words, some states seem to be more attractive for the system than others. States that are visited more often, thus stable and recurrent states, are termed *attractors* (Hollenstein, 2007, 2013). It is easy for the system to rest in these states and more difficult to exit them. Returning to our emotion example, negative mood or even depression have been discussed as attractors (Johnson and Nowak, 2002). Looking at organizational teams, a team leader might constitute an attractor because the conversation among the team tends to center around him/her during an interaction episode such as a team meeting. Likewise, a team with a history of conflicts might fall back into accusatory patterns as soon as certain themes are mentioned in a meeting. The opposite of attractors are *repellors*, states that are visited less often (Hollenstein, 2007). It is more difficult for the system to reach these states and easier to leave them. As an illustration, the concepts of attractors and repellors are often represented as an undulating landscape of peaks (i.e., repellors) and valleys (i.e., attractors; Hollenstein, 2007). The behavior of the system is traceable like a trajectory or “walking path” as the system moves through the state space.

The arrangement of attractors and repellors is not set in stone. Instead, systems evolve and often adapt to changes in the environment. At certain critical points in time, the system breaks out of its usual pattern and forms new dynamics before stabilizing in a new pattern. This reconfiguration of the state space is labeled *phase transition* (Hollenstein, 2007). An example often used in developmental psychology is puberty. Puberty is characterized by a temporary increase in variability, including entirely new patterns of behavior that teenagers might exhibit. As a result, systems are less predictable during a phase transition (Hollenstein, 2013). After the transition, a new stability matures. For an organizational team, a phase transition might occur when a new team member joins the team, when the team has to take on radically different tasks, or when a major misunderstanding causes conflict among the team members.

## State Space Grids

SSGs are one way to empirically test concepts from dynamic system theory in a very accessible manner (Hollenstein, 2007). The SSG technique allows for the visualization of real-time trajectories and provides various quantifications for the content and structures of these trajectories. In the following, we first describe the general set-up of SSGs and present key studies on the technique. Next, we introduce typical measures that can be derived from the visualization.

### Visualizing Patterns of Dynamic Interactions

The SSG is a graphic representation of the state space of a dynamic system and plots the system’s trajectory as it moves through the state space. Most studies that employ the SSG technique focus on just two dimensions (i.e., variables) that characterize the state space. Like a chessboard, the SSG is then “a two-dimensional plane formed by the intersection of two perpendicular dimensions or axes” (Hollenstein, 2013, p. 11). Each position on the grid can be expressed as a combination of one value on the *x*-axis and one value on the *y*-axis. SSGs can be derived from any categorical dimensions^2^ as long as the values on both dimensions are mutually exclusive and exhaustive so that all possible states of the system are mapped out (Hollenstein, 2013). The scale and/or range of each dimension does not have to be equivalent which means that the state space does not have to be a perfect square (Hollenstein, 2013). It is important, however, that the two dimensions underlying the SSG can be assessed at the same point in time as each cell represents the simultaneous combination of the two values in the corresponding row and column. Thus, the event sequences for the two dimensions need to be synchronized. Any time series with at least two synchronized streams of coded categorical data is suitable for creating a SSG (Hollenstein, 2007).

SSGs as a methodological application rooted in the dynamic systems perspective were first introduced to the field of developmental psychology by Lewis et al. (1999). Today, new developments with regard to the SSG technique and the related GridWare software (see below) are headed by Tom Hollenstein at Queen’s University, Kingston, Ontario. SSGs were originally developed as a novel approach to study dynamic processes in early socioemotional development. Specifically, the initial study by Lewis et al. (1999) focused on infants’ attention to their mothers, measured as their angle of gaze and their simultaneous levels of distress. Infants were observed at two waves, when they were 10–12 weeks old and again when they were 26–28 weeks old. Thus, the technique was originally developed to depict and measure changes in intra-individual dynamics (i.e., the individual as the system). A similar approach can be found in a recent study focusing on the relationship between mood and rumination in remitted depressed individuals (Koster et al., 2015). Granic and Lamey (2002) extended the SSG technique to parent–child interactions (for more recent examples see Ha and Granger, 2016; van Dijk et al., 2017), and most studies that followed focused on dyadic interactions. For example, SSGs have been used to describe teacher–student interactions (for an overview see Pennings and Mainhard, 2016), coach–athlete interactions (Erickson et al., 2011; Turnnidge et al., 2014), therapist–client interactions (Tomicic et al., 2015; Couto et al., 2016), or interactions in romantic couples (Butler et al., 2014; Sesemann et al., 2017). Despite this focus on dyadic systems, we believe that SSGs also provide a powerful tool to describe patterns of dynamic interactions in groups and teams. To illustrate, let us introduce a short example.

**FIGURE 1:**
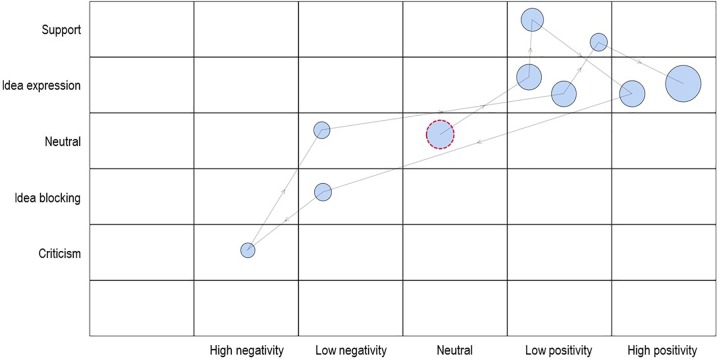
An example of using a state space grid to display the first 10 events of a hypothetical brainstorming session. The team’s energy level is plotted on the *x*-axis and coded talk is plotted on the *y*-axis.

Figure 1 shows a sample SSG for a hypothetical team that is currently brainstorming new ideas. We built this sample SSG using the SSG package implemented in Interact (Mangold, 2017), a commercial software for video annotation. There is also a free software option called GridWare (Lamey et al., 2004) which can be downloaded from www.statespacegrids.org. The website also offers an overview of published studies on SSGs and thus provides an excellent starting point for group and team researchers who are interested in the technique.

The sample SSG in Figure 1 depicts the relationship between coded talk (on the *y*-axis) and the team’s energy level (on the *x*-axis). Please note that this SSG is not based on actual data but serves as an illustration. The verbal interaction was categorized using five behavioral codes, namely, support, idea expression, neutral statement, idea blocking, and criticism. The team’s energy level was coded into five categories, ranging from high negativity, to neutral, to high positivity. The combination of the two dimensions results in a grid with 25 individual states. By default, the software adds an additional row (at the bottom) and column (far left).

The behavioral trajectory (i.e., the sequence of states) is plotted as it proceeds in real time. In this particular example, we coded a total of 10 consecutive events. Each circle (also called node) represents a joint occurrence, and the size of the circle denotes the duration of each particular event. The larger the circle, the longer the two corresponding codes were logged for that particular time unit. The placement of the circles within each cell is random and can be manually adjusted as needed. The red bordered circle denotes the first joint occurrence of coded talk and coded energy. The colors can be adjusted to one’s preferences. This first event shows that the team started the brainstorming session with a neutral statement that was also neutral in tone. The arrows connecting the circles represent the order of the events. Hence, the second statement was coded as an idea put forward in a low positive tone, and so forth. In general, the idea and support statements in our example were accompanied by a positive energy level, whereas statements that were coded as idea blocking or criticism were associated with low to high negativity. Thus, the team in our example did not (yet) visit all the states in the SSG.

### Quantifying Patterns of Dynamic Interactions

In addition to being a visualization tool, SSGs can be used to derive various measures that describe the dynamics of the observed system. Which measures are ultimately used to further quantify the SSG depends on the specific research questions at hand. The original GridWare software provides more measures to choose from than the SSG application in Interact, which is why we used both. In the following, we want to give an overview of those measures that are frequently turned to in SSG studies. These measures can tap both the *content* and the *structure* of the dynamic system (e.g., Granic and Hollenstein, 2003; Pennings and Mainhard, 2016).

Starting with content, the most straightforward approach is to focus on frequency measures and use this information to explore possible attractors and repellors. Thus, content measures can help to identify which states were visited most or least often. In our example above, we can see that three states were visited twice, four states were visited once, and 18 states were not visited at all. There is an important distinction between *events* and *visits* when it comes to SSG measures. Whereas events refer to any node visible in the SSG, a visit is always a transition from one cell to the next. The number of visits therefore provides information about the variability, that is the degree of state transitions, of the system. We will come back to this point when turning to the measures that capture the structure of SSGs. In our sample trajectory in Figure 1, with every event the system transitioned to a new cell. Therefore, we count 10 events and 10 visits. We chose this set up for simplicity but, of course, events can also occur consecutively within one cell. In such cases, the number of events is greater than the number of visits. In addition to raw frequencies, percentages may be considered to aid the comparison across different trajectories (or teams). Another way to standardize frequency measures is to divide them by the total duration of the trajectory. When SSGs are based on real-time recordings (i.e., moment-to-moment dynamics) and an adequate software solution was used to annotate the interaction data (i.e., including time stamps), researchers can obtain measures for duration in addition to frequency.

Based on how often and how long interaction was located in a specific cell, there are different ways to locate attractors and to describe their stability. While some approaches are more descriptive in nature, others require more intensive modeling. The respective procedure also depends on whether attractors are to be empirically identified bottom-up or whether they are derived from theory (Hollenstein, 2013). A simple way to describe attractors is to focus on those cells with (a) the highest number of visits, (b) the highest total duration, or (c) the highest mean duration per visit (Hollenstein, 2013). Such measures are not necessarily rigorous enough to provide a solid attractor analysis, but they are a good first step. If researchers are interested to explore which states actually have a higher probability of occurrence, then the *winnowing procedure* described by Lewis et al. (1999) might be suitable. This iterative step-by-step procedure first deletes those cells with the lowest duration. Next, a heterogeneity score is computed for each cell based on the observed and expected duration for each cell. As such, the winnowing procedure shares common ground with chi-square tests of independence. Interested readers are referred to Hollenstein (2013) who provides a detailed description of the method.

Once one or several attractors, or repellors, are identified, additional measures to describe their stability or strength can be used. The *average return time* to a specific cell or region describes the “pull” of the attractor. Shorter return times indicate that the system only temporally moves away from the attractor but then returns quickly, whereas longer return times may be an indication of a weaker attractor. Similarly, the total number of discrete visits to any other cell before returning to the attractor (i.e., *mean return visits*) describes the strength of an attractor, this time in terms of frequency and not duration.

The measures for attractor strength demonstrate that a dynamic system always wanders around the state space to some extent. In fact, the system would not be dynamic if it were “stuck” in only one particular state. Hence, measures of structure are important to describe the variability and patterns of the observed system. In the following, we want to briefly touch on the following four measures of structure, which we find especially suited for describing dynamic team interactions, namely (a) cell range, (b) total cell transitions, (c) dispersion, and (d) entropy.

*Cell range* is the total number of cells visited by the system. In our example in Figure 1, only seven out of 25 possible cells or states were visited. Hence, 72 percent (i.e., 18 cells) of the state space remains unexplored at this point in time. Of course, it is important that there is sufficient data for interpretation. Since we only included 10 data points in our example, it was physically impossible for the system to visit all states. Of the four variability or structural measures presented, cell range is the least dynamic measure.

*Total cell transitions* comprises the number of visits to the next cell, and therefore describes how intensely the system moves from state to state. Because the very first visit is not counted as a transition, the number of transitions between cells is expressed as the number of visits minus 1. In our example, the system always moved to a new cell with each time step. Hence, the total count of cell transitions is 9. Researchers interested in using this measure should attend to how they conceptualize transitions from cell to cell (Hollenstein, 2013). A total of 9 transitions, for instance, could have occurred between seven cells as in our example or between just two cells such that the system switched back and forth between two states. Thus, the number of cell transitions can be high even though the cell range is rather low. This also shows that in most cases it is useful not to look at certain SSG measures in isolation, but to use several measures simultaneously to describe the grid.

*Dispersion* is a measure that describes how much the coded events are scattered across the state space, controlling for relative duration. Its calculation is based on the number of visited cells and their duration. Mathematically, it is “the sum of the squared proportional durations across all cells, corrected for the number of cells” (Hollenstein, 2013, p. 46). The measure is inverted to reflect numbers between 0 and 1. Higher values indicate a higher variability, thus less rigid interaction. A value of zero would mean that all interaction took place in just one cell. A value of 1 would mean that interaction occurred evenly spread across all cells. In our example, dispersion reached a value of 0.84. Although the values are standardized and are in the range of 0–1, a comparison across different SSGs is particularly useful if their underlying dimensions are the same.

*Entropy* is a measure of predictability and describes the level of organization of the system. In GridWare entropy can be calculated based on cell visits (i.e., visited entropy), cell transitions (i.e., transitional entropy), and duration (i.e., duration entropy). To clarify, consider the following sequence of coded behavior ABABABAB with A and B being discrete codes, such as a joint occurrence of idea expression and low positivity. This particular sequence is much easier to recreate than the following sequence, ACBFDAAB, which seems rather random. For computing entropy, a conditional probability is calculated for each cell. For example, the probability of visiting cell A is calculated by dividing the number of visits in cell A by the total number of visits. These individual probabilities are then summed up for the entire grid based on the formula by Shannon and Weaver (1949). Lower entropy values indicate a highly organized pattern, whereas high entropy denotes unpredictability. The exact formula and implementation in GridWare is described in Hollenstein (2013; see also Dishion et al., 2004). In our example, visit entropy was 1.89. The interpretation of this measure should be based on the respective study and the structure of the SSG. For example, a comparison across different teams who have worked on a similar task and whose interaction were analyzed with the same coding system would likely yield interesting insights.

Of note, the SSG technique offers a range of measures and, although tempting, these measures should not be used blindly in subsequent analyses. Instead, the choice of a specific SSG setting and accompanying measure in GridWare or Interact software should be guided by theoretical considerations.

## Benefits and Implications for Team Science

Team interactions are dynamic and can be rather messy (e.g., Cronin et al., 2011). Adopting a fine-grained behavioral approach to investigate team interactions typically generates large amounts of data that can be difficult to make sense of (e.g., Kozlowski et al., 2015). The SSG technique can address this challenge and innovate the study of team evolution and maturation processes. In the following, we first describe the strengths of the SSG approach before we outline how this technique complements existing analysis strategies.

### Strengths of the SSG Approach

The strengths of the SSG approach to innovate team science broadly fall into three areas. First and foremost, the conceptual approach underlying SSGs can innovate team science by applying non-linear dynamic systems theory and changing the epistemology of teams (for a detailed discussion, see Ramos-Villagrasa et al., 2018). The opportunity afforded by SSGs of embracing the notion of teams as complex and dynamic systems and moving away from the typical linear thinking that has predominated team research (cf. Ramos-Villagrasa et al., 2018) is particularly fruitful for advancing our understanding of the evolution and maturation of teamwork and team processes. Team interactions can be chaotic and teamwork may move in spurts rather than flow evenly toward team outcomes. This is particularly true for teamwork in the face of trends toward increasing team fluidity and temporary organizing (i.e., quick changes in team composition), distributed teamwork (i.e., members collaborating from a distance and interacting and coordinating their actions in intervals), and multiple team memberships (i.e., employees finding themselves in different roles across different teams). In light of such developments, teams are discussed as “dynamic hubs of participants” rather than clearly bounded structures (Mortensen and Haas, 2018). We expect that the interactions that ensue in these dynamic hubs are even less likely to follow linear rules than in traditional teams, and SSGs can account for this possibility.

The second strength of SSGs constitutes visualizing team interaction patterns and making complex team dynamics more accessible. This can be tremendously helpful especially for exploratory research stages, for example when there is little or no prior empirical research on team dynamics and team interactions in a particular team setting. As discussed by Granic and Hollenstein (2003), SSGs can summarize complex interactional data in an intuitively appealing manner (Granic and Hollenstein, 2003; Pennings and Mainhard, 2016). Whereas the theoretical underpinnings of dynamic systems theory may seem daunting, the visualization of such system dynamics via SSGs helps team researchers grasp the characteristics of the team as an interacting system from a holistic perspective. Visualizing the complexity of team interactions may be particularly helpful for understanding team contexts that involve frequent changes or “upheaval” and that require teams to develop swift trust and rapid collaboration (i.e., quickly settling into new routines). This includes action teams (e.g., first response teams) as well as agile teams (e.g., software development teams), where behavioral interaction patterns emerge quickly and where teams are often characterized by fluidity and low stability in team boundaries (Mortensen and Haas, 2018). In those contexts, the adoption of dynamic systems theory for team science will be particularly fruitful, and SSGs as a visualization tool can help position and guide the scholarly thought process in this regard.

When utilizing SSGs as a visualization tool, it is important to decide how to best arrange the different categories along the two axes of the grid. Rearranging the categories may be very helpful for “reading” the interaction more intuitively but should align with the theoretical underpinnings of the respective study. Moreover, the use of SSGs as a visualization tool for complex team interaction dynamics also incorporates a movie function that allows the inspection of a team trajectory evolving over time (see Hollenstein, 2013). Team researchers can either explore the cumulative trajectory of an overall observed team interaction, or they can select specific time windows for shorter trajectories (e.g., for highlighting particularly eventful or critical episodes within a longer stream of team interaction). While this analysis remains qualitative, it can facilitate more dynamic theorizing about the evolution and maturation of team processes. Furthermore, the visualization of complex team dynamics via SSGs may generate innovative research hypotheses to be tested in further analyses.

The third strength of the SSG approach concerns novel opportunities for empirical research and hypothesis testing based on the quantitative measures for complex interaction patterns derived by SSG software. SSGs provide a wide array of different measures that can be compared to traditional measures or added to existing models. Measures cannot only be obtained in a cumulative fashion, as in our example above, but also for smaller time slices within a larger data set. For example, we could request the number of events per cell for every 5 min of an observed team meeting interaction and thus obtain information about the dominant speaker (or any other measure of interest) for each temporal slice of interest. Such an approach opens up new possibilities for investigating how team processes evolve at a quicker pace and within much smaller time frames than typically investigated in temporal team process research, and departs from larger-scale temporal frames for conceptualizing team emergence (e.g., Kozlowski, 2015).

Relying on the SSG technique to quantify team interaction dynamics may be especially useful in the context of infrequent or rare team interaction behaviors. When applying a quantitative behavioral observation approach, team researchers may feel inclined to neglect such behaviors given their low base rate, or choose to combine them with other behaviors in order to obtain more frequent categories (see Lehmann-Willenbrock and Allen, 2018, for a more detailed discussion of decisions to be made when coding team interactions). The SSG technique is sensitive to such low frequency behaviors, which are sometimes highly informative (e.g., when a rare behavior only occurs in successful but not in unsuccessful teams).

As a guiding reminder, team researchers looking to apply SSGs to study team interaction dynamics need to be aware and make informed decisions about how their approach to coding the observed data will affect the results regarding system dynamics that can be obtained using the SSG technique. Of note, this does not necessarily mean that SSGs are applied to evaluate entire theories, but rather refers to making conceptually sound decisions about the operationalization of relevant team constructs at the behavioral event level. Decisions about how relevant team interaction phenomena can adequately be captured in terms of observable behavioral units should be guided by conceptual arguments (cf. Lehmann-Willenbrock and Allen, 2018), which also applies to decisions about SSGs. In other words, when choosing SSGs to quantify interaction dynamics, team researchers need to be mindful when conceptualizing the state space to ensure that those phenomena or variables of interest that will later fall onto the two dimensions of the grid will be assessed at the same time. Moreover, especially when measures of duration are of interest to a researcher, clear unitizing rules are imperative (i.e., deciding when each behavioral unit within the temporal team interaction stream starts and ends).

### Complementary Analyses

The SSG technique shares common ground with some other analytical strategies that aim to distil higher-level emergent patterns from lower-level interaction among individual elements. Thus, we do not want to position SSGs as the new “holy grail” of team research. To put it in the words of Hollenstein (2013, p. 108), “[SSG] are an important tool but often it takes many tools to complete the understanding of the phenomenon at hand.” We have identified two techniques that, in our opinion, are useful complements to the analysis of SSGs, specifically recurrence quantification analysis (e.g., Eckmann et al., 1987; Webber and Zbilut, 2005; Knight et al., 2016) and sequence analysis (e.g., Bakeman and Quera, 2011; Herndon and Lewis, 2015; Klonek et al., 2016). In the following, we briefly compare the main similarities and differences between the SSG technique on the one hand and recurrence quantification analysis and sequence analysis on the other hand, respectively. Readers interested in an overview of additional methods for pattern recognition in team process data are referred to Poole (2018) or Ramos-Villagrasa et al. (2018).

As described earlier, SSGs are a tool for visualizing and quantifying the trajectories of categorical time-series data such as coded team interactions. Turning to team interactions during organizational meetings as an example, researchers may ask questions such as: Does team behavior A typically coincide with team behavior B? Do certain behavioral pairings occur more often than others? Is the interaction evenly distributed across the state space (i.e., flexible patterns) or “boxed” into specific corners (i.e., rigid patterns)? Is each team unique in terms of exhibiting qualitatively different patterns (e.g., distinctive trajectories resulting in idiosyncratic attractors) or can we identify similarities in interaction patterns across different teams?

Another non-linear approach based on the visualization of time-series data is recurrence quantification analysis (Eckmann et al., 1987; Webber and Zbilut, 2005). The visualizations at the heart of this approach are called *recurrence plots* (Marwan et al., 2007; Marwan, 2011). In its most classical application, a recurrence plot spans two dimensions, but shows the same time series on both axes (e.g., ABACABC, with A, B, and C denoting discrete behavioral codes). In contrast to a SSG visualization, the recurrence plot does not show specific values along the two axes, and the plot does not become denser with time as more and more events are entered. Instead, the recurrence plot shows when a specific value in the time series repeats itself (e.g., the code “A” reoccurs at positions 3 and 5) and the plot itself gets larger when the time series is longer. Whenever there is a repetition in the time series, these recurrence points are marked black in the recurrence plot (Marwan, 2011). The basic idea underlying the use of recurrence plots is that researchers can recognize repetitive sequences in the time series with the naked eye, which resembles the basic notion of SSGs. Similarly, recurrence quantification analysis offers various measures that can be obtained from the visualizations such as the percentage of recurrence (Webber and Zbilut, 2005).

Since recurrence quantification analysis typically focuses on the repetitive properties of a dynamic system within itself, this method may seem less intuitive to team researchers at first glance (but for previous applications in team science, see Ramos-Villagrasa et al., 2012; Knight et al., 2016). Moreover, recurrence quantification analysis focuses exclusively on the *structure* of a system’s dynamics; implications regarding the content of the system dynamics are limited. Results of this type of analysis need to be interpreted within a precisely elaborated theoretical context. Consequently, recurrence quantification analysis is less suitable for exploratory research stages. Sample research questions when applying recurrence quantification to coded team meeting interactions could include: does the team show structural recurrence in interaction data or are their interaction patterns chaotic? Are repetitions in behavior more apparent at the beginning or end of the meeting? Are there breakpoints during the meeting after which the interaction is more/less structured? How complex are the detected recurrence structures?

A benefit of recurrence quantification analysis concerns its ability to process continuously sampled signals (e.g., physiological data). When working with continuous measures, researcher need to specify a recurrence threshold (i.e., specifying when an event is marked as recurrent), which illustrates that the method is mathematically more demanding than an analysis based on SSGs as it includes finding optimal parameters (Marwan, 2011). In sum, we would argue that the SSG technique is to some extent more accessible for team researchers than recurrence quantification analysis, even though the two methods build on similar ideas—both conceptually and methodologically. We are not aware of any studies that use a combination of both techniques, but we certainly consider this promising (see also Hollenstein, 2013).

Another methodological approach to the study of team dynamics is to focus on and identify “sub-sequences” in coded team interactions (Poole, 2018). Approaches in this tradition explore more immediate temporal contingencies among coded events and can be subsumed under the umbrella term sequence analysis (Quera, 2018). Notably, sequence analysis is not one particular technique but rather “a toolbox of techniques” (Bakeman and Quera, 2011, p. 134). Over the years, different and increasingly advanced procedures for sequence analysis have been developed (Quera, 2018).

The types of research questions that can be explored with sequence analysis include the following: does behavior A trigger or inhibit behavior B, C, or D? Which behaviors A, B, or C increase the likelihood for behavior D? Which behaviors A, B, or C can inhibit behavior D? Most frequently in team research, studies using sequence analysis explore the extent to which team members reciprocate verbally (i.e., does behavior A trigger more of the same). For example, previous research has explored whether complaining leads to further complaining during organizational team meetings (Kauffeld and Meyers, 2009). Other research has utilized sequence analysis to test whether monitoring behaviors trigger different responses in higher- vs. lower-performing anesthesia teams (Kolbe et al., 2014). For such research questions, the researcher needs to specify a specific time lag. Time lags refer to the number of steps that separate a particular behavior from a criterion event. Lag1 refers to a coded event directly following the previous one (e.g., does code B immediately follow code A); lag2 refers to second-order transitions when a coded event is followed by the next but one coded event, and so forth (Bakeman and Quera, 2011). Lag sequential analysis can then test whether a certain sequence of events is statistically meaningful by comparing the observed transition frequencies to those expected by change. In contrast to SSGs, sequence analysis provides a statistical check for the sequential relationships found in the coded data. Although this is certainly also possible with quantifications derived from SSGs, the SSG technique in and of itself is much more descriptive in nature. In fact, this was one of the main reasons for the development of SSGs (Hollenstein, 2013). Sequence analysis is more rigid in comparison to SSGs because it requires the researcher to make specific assumptions about the expected patterns of behaviors. In addition, behavioral contingencies at higher lags are increasingly difficult to model because they require larger amounts of data (Quera, 2018). Yet, “often, meaningful responses in interpersonal interactions are not immediate” (Hollenstein, 2013, p. 109).

A more recent sequential analysis technique that addresses some of these caveats is *time-window sequential analysis* (Yoder and Tapp, 2004; Bakeman and Quera, 2011). Group researchers can use this technique to test whether a certain response occurs within a pre-defined time window such as a 5 s time-window (i.e., a behavior is contingent if we see a response within 5 s; Bakeman and Quera, 2011). From a conceptual point of view, this approach can solve some of the difficulties associated with specifying meaningful time lags. However, its practical implementation is more difficult, since time-window sequential analysis is not integrated in common observational software such as Interact (Quera, 2018).

Likewise, team researchers rarely turn to sequence analysis for exploring co-occurrences in parallel coded strings of events, although there are procedures that allow this (Quera, 2018). As a result, sequence analysis is often used in a simplified form (Herndon and Lewis, 2015). To recall, with SSGs the combination of at least two variables or dimensions is of interest. As such, the two analysis strategies could by combined by using the observed co-occurrences revealed with the aid of SSGs as a basis for a subsequent sequence analysis. In return, SSGs could be used to visualize the results obtained from sequence analysis and make the findings more tangible.

Finally, despite its many advantages and application possibilities, sequence analysis is not particularly sensitive to low frequency behaviors (for a detailed discussion of the limitations of the sequence analysis approach, see also Chiu and Khoo, 2005). Common practice is therefore to collapse fine-grained categories into larger macro codes and/or to pool the data across groups in order to base the analysis on a larger number of codes (e.g., Klonek et al., 2016). However, this approach regards groups as largely homogeneous, which has been criticized as a simplistic reductionist view on teams and team processes (Hewes and Poole, 2012).

In sum, the SSG technique has much to offer for team science. To date, SSGs have mainly been used for studying interactions in dyadic settings, outside the realm of team science (e.g., Pennings et al., 2014; Guo et al., 2017). We hope that team researchers will begin to embrace the SSG technique for enabling novel insights into the complex interactional dynamics at the core of team functioning and performance (e.g., Ramos-Villagrasa et al., 2018).

## Application Example and Tutorial

To make the application of SSGs more tangible to team research and development, we will now present an example based on real team data. We provide step-by-step suggestions for using the technique and hope to highlight the various opportunities that SSGs offer.

### A Step-by-Step Overview

As we have pointed out above, researchers should not begin considering SSGs in the final stages of an investigation. Rather, the decision to employ SSGs should be made early in order to be able to account for the requirements of this technique. In Table 1 we have summarized the key steps for using SSGs in team research and development.

**Table 1:** Basic steps for applying SSGs in team research.

Basic steps	Considerations
(1) Define the (research) aim	- Clarify how the context and purpose of the study is linked to the dynamic systems perspective.
	- Describe the theoretical fundaments for temporally sensitive interaction dynamics.
	- Identify the underlying dimensions of the state space.
(2) Define phenomena and variables of interest	- Decide how the state space is constructed and define the variables of interest.
	- Variables must be observable simultaneously.
	- The variables should be mutually exclusive and exhaustive.
(3) Select unitizing rule (e.g., turn of talk)	- Units for the variables observed should be measured at the same time intervals.
	- Preferably, time units should not be smaller than 1 s.
(4) Choose existing coding scheme(s) or develop a new one	- Chose or develop one or several coding schemes that fit the research question.
	- A smaller number of categories will yield a better overview.
(5) Gather interaction data and code the data	- Record data such that the variables of interest can be measured effectively.
	- Train coders and establish inter-rater reliability.
(6) Visualize and quantify data in regards of the research question	- Create a SSG for each team using Interact (Mangold, 2017) or GridWare (Lamey et al., 2004) software.
	- Interpret the SSGs and derive adequate measures from the visualizations.
	- Several types of analyses can be conducted on the measures the software offers.
(7) Provide feedback to the team	- Chose a format that communicates the contents of the analysis which are relevant feedback for the target recipients.

*Answers were provided on a Likert scale ranging from 1 = very low to 5 = very high. ^a^Schippers et al. (2007), ^b^Rogelberg et al. (2010), ^c^Carless and De Paola (2000), ^d^Jehn (1995).*

The first step involves defining the research aim and identifying the theoretical foundations for capturing team phenomena at the behavioral event level and specifying temporally sensitive interaction dynamics in the study context. The two chosen variables should be meaningfully related and their interaction should be grounded in theory. Most likely, the nodes or data points (i.e., the observed behavioral units) will not be randomly scattered across the state space but organized into clusters. It is advisable to find theoretical support for grouping the expected patterns of nodes into meaningful clusters. Hence, theory-based considerations should drive how a SSG is structured, and how this relates to the overarching team phenomenon that is studied. This step will ensure an early integration of the SSG technique as a methodological tool into the concept of the study.

The second step entails defining the variables of interest. Since the variables need to fulfill specific norms to be used for SSG analyses, it is imperative to account for such norms early on as well. In particular, it is important that the chosen dimensions underlying the SSG can be observed and coded in a sequential fashion (i.e., moment-to-moment). Likewise, the dimensions should be constructed in a way that they allow for mutually exclusive and exhaustive coding. It is therefore important to choose two variables that have similar granularity.

Closely related, the third step includes that both variables need to be unitized identically. For instance, if one variable was measured every 2 min (e.g., mood), the second variable (e.g., number of solutions mentioned) has to provide a data point for every 2 min as well. Hence, this aspect is important to consider at the research design stage, when making decisions regarding the operationalization of variables. The chosen software may pose additional requirements. For instance, the smallest time scale GridWare processes are seconds. Missing data should be avoided as this interrupts the interaction flow and thus the trajectory.

In the fourth step, an appropriate coding scheme can be chosen or developed. Available fine-grained coding schemes may be adjusted and summarized into broader categories to fit the purpose at hand. Note that each dimension (variable) may be coded with a different scheme (e.g., verbal and non-verbal interaction). Although it is not a theoretical requirement, for practical reasons a smaller number of coding categories, for example six to eight on each dimension, will yield a better overview and serve the purpose of applying SSGs as an analytical and/or visualization tool.

In the fifth step, once all these decisions have been taken, behavioral process data (video/audio recordings or live coding) can be gathered and coded. It is worth ensuring high-quality data through appropriate training of coders and establishing inter-rater reliability. Depending on the sample population, questions around data storage and privacy policies should be clarified before data collection and coding.

In the sixth step, once the coding is completed and visualizations are available for each team, the SSGs can be interpreted and appropriate measures for describing both the content and structure of the trajectories can be calculated. These measures can be easily exported and used for further analysis in other statistic software programs.

Finally, beyond research purposes, the coded data may be used for team development as detailed below. The visualizations, even more so than the measures, can serve as a basis for feedback.

### The Data Set

Data for this application example were sampled from a recently gathered data set that has not been published to date. The data set comprises videotapes of the first (T1) and the final (T2) team meeting of a 6-week long student project at a large Dutch university. The project resembled the work of organizational consultants and required the teams to develop a managerial strategy for an organizational change project. The study was approved by the Economics and Business Ethics Committee at the University of Amsterdam. Participation in the study was voluntary, and all participants provided their written informed consent. From this pool we selected two five-person teams with roughly equal meeting durations on the basis of their productivity (high vs. low). On average, these four team meetings lasted for 55.14 min (*SD* = 4.08). As a proxy for productivity, we took the rate of solutions mentioned per hour. The productive team produced 19.45 solutions per hour at T1 and 21.15 solutions per hour at T2. The unproductive team produced 6.94 solutions per hour at T1 and 9.66 solutions per hour at T2. As shown in Table 2, the productive team consistently scored higher on positive team characteristics like reflexivity, cohesion, and meeting satisfaction and lower on team conflict measures.

**Table 2:** Aggregated scores on team characteristics for each team at T1 and T2.

Team characteristic	T1		T2	
	Unproductive	Productive	Unproductive	Productive
Reflexivity^a^	3.35	4.35	2.85	4.05
Meeting satisfaction^b^	4.13	4.87	4.10	4.50
Social cohesion^c^	3.20	3.80	2.83	4.00
Task cohesion^c^	4.47	4.87	4.27	4.67
Intragroup conflict (relationship)^d^	1.45	1.05	2.00	1.05
Intragroup conflict (task)^d^	2.10	1.55	2.70	1.50

### Formatting the Data

We coded the observed team meeting interaction using the act4teams coding scheme (e.g., Kauffeld and Lehmann-Willenbrock, 2012; Kauffeld et al., 2018) and Interact software (Mangold, 2017). Act4teams is a mutually exclusive and exhaustive coding scheme for measuring problem-solving dynamics that occur in groups and teams. Using the act4teams coding scheme, a behavioral code is assigned to each verbal thought unit, which is typically a single sentence. In order to reduce complexity, we collapsed the 43 fine-grained act4teams codes into six broader aspects of interaction. These covered elements of interactions that were knowledge-oriented, problem-focused, structural, action-oriented, relational, and counterproductive. To ensure that the coding was exhaustive, we included an additional filler code labeled “other behavior.” An overview of the simplified coding scheme including sample statements for each code is shown in Table 3. With each coded statement, we also recorded who the speaker was. Thus, our data format meets the requirements for SSGs explained in section “Visualizing Patterns of Dynamic Interactions.” The coding leads to a multivariate time series of sequentially coded categorical data.

**Table 3:** Behavioral categories, descriptions, and sample statements.

Behavioral category	Description	Examples
Knowledge-oriented	Sharing organizational knowledge, referring to experts, and asking questions about opinions, content, or experience.	“Well, I format it like this …”, “The guidelines are on blackboard.”, We should ask Marisa about that.”
Problem solving	Identifying, describing, and analyzing problems and solutions.	“We have not yet clarified the concept.”, “We have to narrow our focus.”, “We should stick to the marking guidelines.”
Structural	Structuring the conversation by clarifying, summarizing content as well as structuring the procedure in terms of goals and priorities, time management and task distribution.	“So in sum, we have to start with this point.”, “Let me write that down.”, “This is a key aspect.”, “We still have 15 min.”
Action-oriented	Showing interest in change and new ideas as well as taking responsibility and planning concrete steps.	“I am curious about the results.”, “That will bring us ahead.”, “Okay, I will research that.”, “I will do that next week then.”
Relational	Positive socio-emotional behavior such as humor, involving and supporting other team members as well as appreciating their contributions.	“If that’s okay with you, Jim.”, “Yes, exactly.”, “Hmm, yes.”, “I have understood that.”
Counterproductive	Behavior which disrupts the productivity of the team such as complaining, denying responsibility or side conversations and self-promotion.	“If everyone did it my way …”, “We will wait and see.”, “What if that ends up nowhere?”, “Mark should have prepared that.”
Other	Behavior which does not fit in any of the previous categories (e.g., pauses, incomplete or incomprehensible sentences).	

**FIGURE 2:**
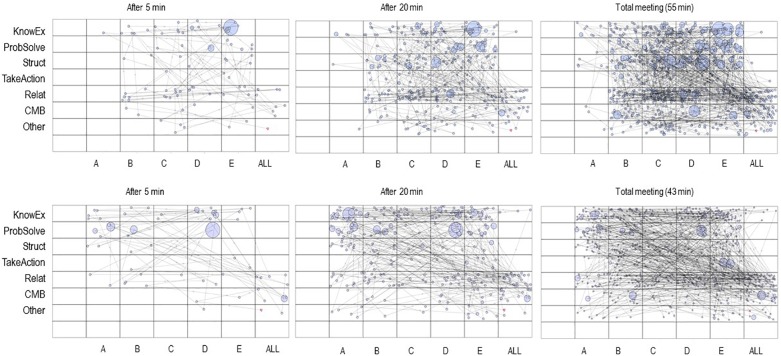
State space grids (SSGs) representing verbal team interactions for a productive and an unproductive team at three time points for the first meeting. The **(top)** three panels show the SSGs of the productive team. The **(bottom)** three panels show the SSGs of the unproductive team. A, B, C, D, and E label each of the five team members per team. The size of the circles denotes the duration of each event. KnowEx, knowledge exchange; ProbSolve, problem solving; Struct, structuring; TakeAction, taking initiative; Relat, relational; CMB, counterproductive meeting behavior; Other, verbal behaviors that do not fit any of the six functional categories.

**FIGURE 3:**
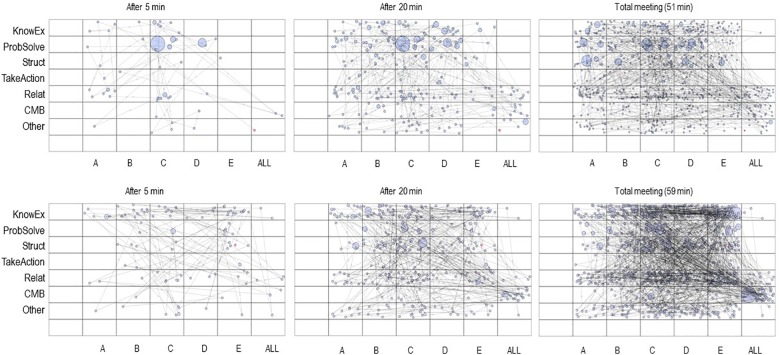
State space grids (SSGs) representing verbal team interactions for a productive and an unproductive team at three time points for the last meeting. These are the same teams as in Figure 2. The **(top)** three panels show the SSGs of the productive team. The bottom three panels show the SSGs of the unproductive team. A, B, C, D, and E label each of the five team members per team. The size of the circles denotes the duration of each event. KnowEx, knowledge exchange; ProbSolve, problem solving; Struct, structuring; TakeAction, taking initiative; Relat, relational; CMB, counterproductive meeting behavior; Other, verbal behaviors that do not fit any of the six functional categories.

Again, we used the SSG application in Interact software for visualization and GridWare software to further analyze the coded team data. Each cell in the grid represents a distinct interactive state defined by the mutual occurrence of a specific speaker (*x*-axis) and the corresponding verbal behavior (*y*-axis). To visualize how the interaction unfolds over the time of a meeting, we created three plots per meeting for each of the two teams (see Figure 2, 3). This is possible through a function integrated in both software applications, i.e., a time slider allows us to choose specific time ranges of interest within the recorded time. The SSG then builds up gradually. The SSG measures can also be calculated for each of the individual time intervals. The plots in Figure 2, 3 depict the interaction trajectory for the first 5 min, for the first 20 min, and for the entire meeting, respectively.

In the following we will discuss the grids and the quantitative measures with regard to the two teams in a more generalized way and point out benefits for both team research and team development where relevant.

### Visual Inspection

Figure 2 shows the developing SSG for the two teams during their initial meeting. At first inspection of the entire meetings, we can observe clear differences between them. Starting with the columns (i.e., speakers), we can see an interesting difference concerning the length and distribution of speaker turns. First, there is a clearer pattern of cells that are visited more often than others in the productive team compared to the unproductive team. Second, more circles in the productive team are larger which indicates longer lasting contributions. Third, the distribution of circles across columns (speakers) in general and that of large circles in particular reveals that in the productive team speakers do not seem to have an equal share in the amount and length of their contributions. Some (speakers D and E) dominate the interaction and others (speaker A) are rather quiet. In the unproductive team the differences between speakers are more difficult to characterize. It seems that the conversational floor is more equally shared.

Turning to the rows and looking at the functional interaction categories, more differences arise. In the productive team, the distribution of circles in the rows shows that some are visited more frequently than others. For instance, cells on the structural level (e.g., clarifying, prioritizing, and time management statements) are visited more often than cells on the action-oriented level (e.g., interest in change and action planning). Again, the unproductive team lacks such a clear trend. Finally, in the productive team we see a dark horizontal shade across the relational level. The shade indicates intensive interaction within that level, that is relational contributions are often followed by other relational contributions. These observations are relatively rough but they provide an overview of the interaction and thus an accessible form of feedback that can be insightful for team leaders and team members themselves (e.g., Who dominates the conversation? Who tends to structure the meeting? Who takes action? What contributions occur at what point during the meeting?). Before turning to the quantification of these observations, we will briefly examine the plots that represent earlier interaction stages within the same meetings. After 5 min, in both teams one individual seems to dominate the interaction: in the productive team, member E makes a number of contributions and a particularly lengthy knowledge-oriented one. This active role seems to remain stable across the meeting. In the unproductive team, after 5 min, member D has a similar role with a prominent problem-solving contribution. D, however, does not remain dominant throughout the meeting. Further, in this first grid the productive team shows more relational interaction compared to the unproductive team. This pattern intensifies throughout the meeting. The unproductive team, however, shows pronounced interaction on the knowledge-oriented level after 5 min that increases over time. To conclude, the two teams show specific and different trends from the beginning, and these may explain higher or lower productivity. Such conclusions highlight the potential of identifying dysfunctional processes early on during the meeting to be able to correct them guiding the team into more productive dynamics.

Figure 3 represents the equivalent interaction trajectories for the final meeting. The patterns for each team look rather different compared to the patterns for the first meeting. For instance, observing the final grid for the productive team, it is less easy to identify a dominant speaker, members seem more equally involved in the interaction compared to the first meeting. Especially team member A who was very quiet at T1 is now fully integrated in the interaction at T2. Circles in the top three rows are larger than in the bottom rows. Thus, knowledge-oriented, structural, and problem-solving contributions take up more time than other types of contributions in the productive team. The unproductive team shows two dark horizontal shadows, one on the top row suggesting an intensive exchange of knowledge-oriented contributions, and one on the relational level indicating strong positive socio-emotional exchange.

### Quantitative Inspection

For many of these observations we can obtain quantitative measures. These help to analyze the content and structure of the interaction within and across grids. In practical terms, it means that we could establish dominant speakers, dominant interaction categories or characterize speakers with regard to their types of interactive contributions. In addition, we can quantify if the interaction was rigid or flexible such that structural patterns in the trajectory can be identified. For example, if we want to know who of the speakers dominated the interaction we can look for the number of events that we find within that speaker’s column or we might look at the proportion of the total time taken up by the events of that speaker. Taking the example of the productive team at T1 (Figure 2) makes clear how critical it is to determine these measures beforehand and rooting this decision in theoretical grounds: considering the number of events per speaker yields member C as the dominant individual (226 events) while we can record much less events for speaker D (141 events) and speaker E (153 events) which we had identified as dominant through our visual inspection. Considering the proportion of the total time per speaker results in a different conclusion: the contributions of the three speakers are rather similar, although speaker E slightly dominates the conversational floor (*C* = 23.5%, *D* = 23.6%, and *E* = 27.3%). Overall, the standard deviation for these percentages was 9.23. Looking at the unproductive team, the standard deviation for the proportion of the total time per speaker was 5.27. This supports our preliminary conclusion about a more even distribution of speaker contributions in the unproductive team at T1. Still, interesting differences exist. Specifically, speaker E’s contributions composed 23.2% of the overall conversation whereas speaker C only contributed 7.1%.

Turning to measures of structure, findings reveal that all teams rather exhibit flexible interaction. The teams explored large parts of the grids with an average cell range of 38.75. Likewise, and because all team members did contribute to the discussion, the values for dispersion ranged between 0.97 and 0.98. Values for visit entropy were in the range of 3.22–3.49. Taken together, these values indicate a highly variable interaction style and show that interaction is rather difficult to predict. Contrary to other studies with SSGs (e.g., van Dijk et al., 2017), our coded team data was not boxed into a specific corner of the SSG. This is not necessarily characteristic for team interaction patterns in general but is, in part, due to how we defined the dimensions in our particular example with speakers on one axis and coded talk on the other.

## Benefits and Implications for Team Training and Development

We would like to conclude this article with suggestions and ideas for the practical application of SGGs. Of note, these suggestions require future empirical work to evaluate their actual utility for team training and development. Yet, overall, we foresee multiple benefits of the application of SSGs in the context of team training and development, facilitating team maturation and evolution over time. First of all, getting teams to consider their team as a system of interactions, rather than a collection of people, may inspire novel understanding and insights regarding interdependencies and team dynamics. However, such a perspective can be quite complex and requires a holistic picture of the team interaction space. Visualizing this holistic picture via SSGs and presenting the behavioral feedback to the team can likely serve as a development trigger in this regard (cf. Lehmann-Willenbrock and Kauffeld, 2010). In the following, we point out specific ways in which SSGs might be used for effective delivery and transfer of training and development, along with recommendations for differing team contexts.

Training is considered effective when it produces changes in cognitive, affective, and/or skill-based outcomes (Salas and Cannon-Bowers, 2001), and leads to transfer of learning to the work context (Blume et al., 2010). For instance, a team diversity training may be aimed at enhancing the willingness to cooperate in diverse teams (e.g., affective changes), increasing knowledge regarding the potential benefits and pitfalls of diversity for teamwork (cognitive changes), providing the skills to more effectively utilize the heterogeneity of ideas and perspectives present in diverse teams (skill-based changes), leading to measurable performance improvements (e.g., Homan et al., 2015). In contrast to team training, team development (e.g., team coaching or developmental assignments) tends to be broader in scope and has a longer-time perspective. The skills to be acquired also typically go beyond those required for effectively accomplishing current tasks, jobs, and/or roles (Aguinis and Kraiger, 2009). Yet, boundaries between training and development are fluid, and both show considerable overlap in the principles followed to ensure effectiveness. Therefore, unless specified otherwise, we use both terms interchangeably and assume that both formats can benefit from SSGs in similar ways.

Training and development strategies typically follow several principles to ensure effectiveness (e.g., Salas and Cannon-Bowers, 2001). These entail presenting concepts and information relevant to the participant; showcasing the knowledge, skills, and abilities (KSAs) to be learnt; allowing for practicing the KSAs; and supplying participants with feedback during practicing and on improvements made over time. We believe that the SSG technique is particularly useful to support the feedback element of effective training and development.

The SSGs allow for detailed and visually appealing feedback based on actual behavior. This feedback can support teams in diagnosing the state they are in terms of team processes (e.g., knowledge sharing and utilization), in reflecting on emergent states (e.g., relational conflict), and in improving on important team processes. For instance, teams could receive feedback on their status quo as well as how their status quo has changed over the course of a training or developmental activity. Scholars have argued that feedback tools with a higher temporal resolution are especially suitable for providing developmental feedback (e.g., Rosen and Dietz, 2017). An important advantage of SSGs is that they allow teams and those involved in team training and development (e.g., leaders, trainers, and coaches) to gain an easily accessible overview of micro-level team interaction data that otherwise would be perceived as messy and difficult to grasp. The software’s “movie function,” as described earlier, may further support such practicing and feedback over time, as it adds further visual stimulation to other established forms of presentation (Myer et al., 2013). In addition, as SSGs can be administered repeatedly, (lack of) improvements could be detected, allowing teams to redirect or strengthen efforts if needed.

As SSGs are based on actual behavior, using this technique for feedback purposes might help circumvent validity and fairness issues. Such issues may arise when feedback is based on attributions or interpretations of behaviors, or of attitudes and underlying traits (e.g., by means of a rating scale completed by one’s supervisor or team members, or by means of a supervisor’s forced ranking of members in a team). Furthermore, feedback on relatively stable dimensions (e.g., intellectual ability) does not offer guidance regarding how to improve one’s behavior. Comprehensible feedback based on actual behavior, however, increases the likelihood that feedback leads to improved performance (e.g., Bandura, 1986; Kluger and DeNisi, 1996; Roter et al., 2004).

Besides their role in feedback, SSGs may be used to demonstrate the KSAs to be learnt during training and development, and facilitate subsequent practicing. For example, for more standardized procedures, teams may watch a video-based example of both an ineffective and effective team interaction. This demonstration could be accompanied by SSGs reflecting the respective patterns of observed interactions in the effective and ineffective example. The trainer or coach could then discuss concrete steps to bring the ineffectively interacting team closer to the effectively interacting team. Alternatively, team members could identify ways to approximate the effectively interacting team’s profile. Yet, “it is important to remember that all teams are not equal” (Salas et al., 2017, p. 21). Especially in complex situations, the results of a SSG analysis of a successful team should not necessarily serve as a model for other teams (i.e., “one size fits all”). In such cases, it is particularly important that the trainer or coach stimulates reflection, so that the team members themselves can decide which elements can serve as a model for their own teamwork. Building a shared understanding of successful team interaction patterns is key to make sure that all team members equally benefit from team training with SSGs. This brings us to our next point, i.e., using SSG for team development.

Compared to team training, team development may entail a longer and less formalized process, allowing for more profound and longer-lasting maturation and evolution processes in teams. Less emphasis is given on how a team compares to other teams (e.g., by comparing the team’s current SSG with the average SSG in the department, organization, or branch). Rather, development is concerned with the team’s growth over time (e.g., Aguinis and Kraiger, 2009). We expect SSGs to be helpful in stimulating this growth, as the technique allows for observing the same aspects of a team’s interaction at different points in time. These points in time may demarcate different “life stages” such as at team formation and in the middle and end of a project (cf. Tuckman, 1965; Gersick, 1988) or phases in a team’s performance cycle (e.g., action versus transition phases; Marks et al., 2001). Depending on the exact purpose, it might be useful to employ the same or different state spaces at different points in time. To observe development on a given behavioral pattern, using the same state space is likely to be most suitable. To understand whether teams appropriately deal with the unique demands that differing stages or phases impose, using phase- or stage-specific state spaces might be more insightful. Teams might also seek to improve their phase-specific behavior over time (e.g., by increasing reflexivity in transition phases and improving on coordination in action phases). In this case, using SSGs repeatedly across multiple performance cycles may prove most conducive to continuous learning.

Finally, certain types of teams may particularly benefit from using SSGs as a feedback and development tool. As our application example shows, there are visible differences in the interaction patterns not only between teams but also across different stages in the team’s life cycle (e.g., as determined by the duration of a project). Identifying characteristic patterns for team processes and emergent states embedded in certain stages of a project could help evaluate team processes in a standardized way. This could be especially interesting in and applicable to the context of SCRUM teams. While their project phases are relatively short and contents may vary according to project, the general procedures employed in SCRUM teams follow similar patterns across projects (Schwaber, 1997; Rising and Janoff, 2000). Furthermore, teams undergoing intense training (e.g., in the form of simulations) before entering the performance stage such a crisis or emergency teams, aviation or astronautic crews, or firefighter and special force units may be particularly attuned to benefit from the fine-grained, behavior-based feedback opportunities of the SSG technique. Systematically studying SSGs obtained during training and development in these team contexts may afford the opportunity to extract knowledge on more generic patterns of effective behavior across types of teams.

## Ethics Statement

The study was approved by the Economics and Business Ethics Committee at the University of Amsterdam. Participation in the study was voluntary, and all participants provided their written informed consent.

## Author Contributions

AM developed the original idea for the manuscript, took the lead in writing, and performed the analyses. CH contributed to writing the manuscript and aided in data analysis and interpretation. NL-W and CB collected the data, critically revised the manuscript for intellectual content, and contributed to writing the manuscript. All authors approved the manuscript to be published.

## Conflict of Interest Statement

The authors declare that the research was conducted in the absence of any commercial or financial relationships that could be construed as a potential conflict of interest.

## Footnotes

1 In accordance with much of the existing literature, we use the terms “group” and “team” synonymously.

2 So far, the SSGs technique has been applied primarily to categorical data. An extension to continuously sampled signals is discussed in Hollenstein (2013).
